# Supplementary material for: Improving the Secretory Expression of an α-Galactosidase from Aspergillus niger in Pichia pastoris
Source: PLoS One. 2016 Aug 22;11(8):e0161529. doi: 10.1371/journal.pone.0161529 (PMC4993465; doi:10.1371/journal.pone.0161529)
Supplement: S1 File — Sequences of the primers for constructing the mutant library (Table A); Comparison of the original and optimized aga gene sequences (Fig A); SDS-PAGE (12%) analysis of the extracellular and intracellular proteins of AGA-X33, AGA-KM71H-2, AGA-KM71H-1 (Fig B); and SDS-PAGE (12%) analysis of the extracellular and intracellular proteins of AGA-E, AGA-I, and AGA-P (144 h post-induction in shaking flasks) (Fig C). (PDF) [file pone.0161529.s001.pdf]

Supporting Information

Improving the Secretory Expression of an α-galactosidase from *Aspergillus niger* in *Pichia pastoris*

Xianliang Zheng[1,2,3], Bo Fang[1,2], Dongfei Han[1], Wenxia Yang[1], Feifei Qi[1], Hui Chen[1], Shengying Li[1]*

[1]Shandong Provincial Key Laboratory of Synthetic Biology, and CAS Key Laboratory of Biofuels, Qingdao Institute of Bioenergy and Bioprocess Technology, Chinese Academy of Sciences, No. 189 Songling Road, Qingdao, Shandong 266101, China; [2]University of Chinese Academy of Sciences, Beijing 100049, China; [3]Sino-Danish Center for Education and Research, Beijing, 100190, China.

*To whom correspondence may be addressed. E-mail: lishengying@qibebt.ac.cn

Table A. Sequences of the primers for constructing the mutant library. The Kex2 P1' site codons are shown in bold. (F: Forward primer, R: Reverse primer, the forward primer and corresponding reverse primer are complementary to each other.)

P1' residues	Primer sequences
A	F-GGTATCTCTCGAGAAAAGA**GCT**GCTGAAGCTGAATTCGCAC<br>R-GTGCGAATTCAGCTTCAGC**AGC**TCTTTTCTCGAGAGATACC
C	F-GGGTATCTCTCGAGAAAAGA**TGT**GCTGAAGCTGAATTCGCAC<br>R-GTGCGAATTCAGCTTCAGC**ACA**TCTTTTCTCGAGAGATACCC
D	F-GGTATCTCTCGAGAAAAGA**GAT**GCTGAAGCTGAATTCGCAC<br>R-GTGCGAATTCAGCTTCAGC**ATC**TCTTTTCTCGAGAGATACC
F	F-GGGTATCTCTCGAGAAAAGA**TTT**GCTGAAGCTGAATTCGCAC<br>R-GTGCGAATTCAGCTTCAGC**AAA**TCTTTTCTCGAGAGATACCC
G	F-GGTATCTCTCGAGAAAAGA**GGT**GCTGAAGCTGAATTCGCAC<br>R-GTGCGAATTCAGCTTCAGC**ACC**TCTTTTCTCGAGAGATACC
H	F-GGTATCTCTCGAGAAAAGA**CAT**GCTGAAGCTGAATTCGCAC<br>R-GTGCGAATTCAGCTTCAGC**ATG**TCTTTTCTCGAGAGATACC
I	F-GGGTATCTCTCGAGAAAAGA**ATT**GCTGAAGCTGAATTCGCAC<br>R-GTGCGAATTCAGCTTCAGC**AAT**TCTTTTCTCGAGAGATACCC
K	F-GGGTATCTCTCGAGAAAAGA**AAA**GCTGAAGCTGAATTCGCAC<br>R-GTGCGAATTCAGCTTCAGC**TTT**TCTTTTCTCGAGAGATACCC
L	F-GGGTATCTCTCGAGAAAAGA**CTT**GCTGAAGCTGAATTCGCAC<br>R-GTGCGAATTCAGCTTCAGC**AAG**TCTTTTCTCGAGAGATACCC
M	F-GGTATCTCTCGAGAAAAGA**ATG**GCTGAAGCTGAATTCGCAC<br>R-GTGCGAATTCAGCTTCAGC**CAT**TCTTTTCTCGAGAGATACC
N	F-GGTATCTCTCGAGAAAAGA**AAC**GCTGAAGCTGAATTCGCAC<br>R-GTGCGAATTCAGCTTCAGC**GTT**TCTTTTCTCGAGAGATACC
P	F-GGGTATCTCTCGAGAAAAGA**CCA**GCTGAAGCTGAATTCGCAC<br>R-GTGCGAATTCAGCTTCAGC**TGG**TCTTTTCTCGAGAGATACCC
Q	F-GGTATCTCTCGAGAAAAGA**CAA**GCTGAAGCTGAATTCGCAC<br>R-GTGCGAATTCAGCTTCAGC**TTG**TCTTTTCTCGAGAGATACC
R	F-GGGTATCTCTCGAGAAAAGA**AGA**GCTGAAGCTGAATTCGCAC<br>R-GTGCGAATTCAGCTTCAGC**TCT**TCTTTTCTCGAGAGATACCC
S	F-GGGTATCTCTCGAGAAAAGA**TCT**GCTGAAGCTGAATTCGCAC<br>R-GTGCGAATTCAGCTTCAGC**AGA**TCTTTTCTCGAGAGATACCC
T	F-GGGTATCTCTCGAGAAAAGA**ACT**GCTGAAGCTGAATTCGCAC<br>R-GTGCGAATTCAGCTTCAGC**AGT**TCTTTTCTCGAGAGATACCC
V	F-GGTATCTCTCGAGAAAAGA**GTT**GCTGAAGCTGAATTCGCAC<br>R-GTGCGAATTCAGCTTCAGC**AAC**TCTTTTCTCGAGAGATACC
W	F-GGTATCTCTCGAGAAAAGA**TGG**GCTGAAGCTGAATTCGCAC<br>R-GTGCGAATTCAGCTTCAGC**CCA**TCTTTTCTCGAGAGATACC
Y	F-GGTATCTCTCGAGAAAAGA**TAT**GCTGAAGCTGAATTCGCAC<br>R-GTGCGAATTCAGCTTCAGC**ATA**TCTTTTCTCGAGAGATACC

**Optimized 1**	GCACCCGCAATTGGGGCTTCTAATTCACAGACGATCGTTACGAATGGCACTAGTTTCGCA
**Original 1**	GCACCCGCAATTGGGGCTTCGAATTCACAGACGATCGTTACGAATGGCACTAGTTTCGCA
**Optimized 61**	TTGAACGGCGACAATGTCTCATATCGATTCCATGTCAACAGTACCACCGGCGACTTGATT
**Original 61**	TTGAACGGCGACAATGTCTCATATCGATTCCATGTCAACAGTAGCACCGGCGACTTGATT
**Optimized 121**	TCTGATCATTTTGGTGGTGTCGTCTCCGGCACAATCCCTTCGCCAGTGGAACCTGCTGTC
**Original 121**	TCTGATCATTTTGGTGGTGTCGTCTCCGGCACAATCCCTTCGCCAGTGGAACCTGCTGTC
**Optimized 181**	AACGGCTGGGTCGGCATGCCTGGTCGAATCCGCCGGGAGTTCCCCGACCAAGGCCGTGGG
**Original 181**	AACGGCTGGGTCGGCATGCCTGGTCGAATCCGCCGGGAGTTCCCCGACCAAGGCCGTGGG
**Optimized 241**	GATTTCCGCATCCCCGCCGTTCGTATTCGGGAATCGGCAGGTTATACTGTTAGCGATCTC
**Original 241**	GATTTCCGCATTCCCGCCGTTCGTATTCGGGAATCGGCAGGTTATACTGTTAGCGATCTC
**Optimized 301**	CAATATGTGTCGCACGAGGTGATCGAAGGTAAATACGCTTTGCCTGGCCTGCCTGCCACA
**Original 301**	CAATATGTGTCGCACGAGGTGATCGAAGGTAAATATGCTTTGCCTGGCCTGCCTGCCACA
**Optimized 361**	TTTGGCGATGCGCAGGATGTCACAACTTTGGTAGTCCATCTGTATGACAACTATAGCTCC
**Original 361**	TTTGGCGATGCGCAGGACGCCACCACTTTGGTAGTCCATCTGTATGACAACTATAGCTCC
**Optimized 421**	GTCGCGGCCGACTTGTCATACTCCATATTTCCGAAGTATGATGCGATCGTGAGGAGTGTC
**Original 421**	GTCGCGGCCGACTTGTCATACTCCATATTTCCGAAATATGATGCGATCGTGAGGAGTGTC
**Optimized 481**	AATGTGACCAACCAGGGCCCGGGTAATATCACTATCGAGGCCCTTGCAAGCATTAGCATC
**Original 481**	AATGTGACCAACCAGGGCCCAGGTAATATCACTATCGAGGCCCTTGCAAGCATAAGTATC
**Optimized 541**	GATTTCCCCTACGAAGACCTCGACATGGTCAGCCTCCGAGGCGACTGGGCCAGAGAGGCA
**Original 541**	GATTTCCCCTATGAAGACCTCGACATGGTCAGCCTCCGAGGCGACTGGGCCAGAGAGGCA
**Optimized 601**	AATGTTCAGAGAAGCAAAGTGCAGTATGGCGTCCAGGGATTTGGAAGCAGTACTGGATAT
**Original 601**	AATGTTCAGAGAAGCAAAGTGCAGTATGGCGTCCAGGGATTTGGAAGCAGTACTGGATAT
**Optimized 661**	TCCTCTCACCTTCACAATCCCTTCCTTGCCATAGTAGATCCAGCTACTACCGAGTCGCAA
**Original 661**	TCCTCTCACCTTCATAATCCCTTCCTTGCCATAGTAGATCCAGCTACTACCGAATCGCAA
**Optimized 721**	GGCGAGGCATGGGGTTTCAACCTTGTATATACCGGCTCTTTCTCAGCCCAAGTAGAGAAA
**Original 721**	GGCGAGGCATGGGGTTTCAACCTTGTATATACCGGCTCTTTCTCGGCGCAAGTAGAGAAA
**Optimized 781**	GGATCGCAAGGTTTCACCCGGGCGCTGCTCGGCTTCAACCCGGACCAATTATCGTGGAAC
**Original 781**	GGATCGCAAGGTTTCACCCGGGCGCTGCTCGGCTTCAACCCGGACCAATTATCGTGGAAC
**Optimized 841**	CTTGGCCCTGGCGAGACTTTAACCTCCCCTGAGTGTGTTGCAGTCTACTCGGACAAAGGC
**Original 841**	CTTGGCCCTGGCGAGACTTTAACCTCCCCCGAGTGTGTTGCAGTCTACTCGGACAAAGGC
**Optimized 901**	CTTGGCTCAGTGTCTCGCAAATTCCACCGGCTATATCGTAACCACCTCATGAAGAGCAAG
**Original 901**	CTTGGCTCAGTGTCTCGCAAATTCCACCGGCTATATCGCAACCACCTCATGAAGAGCAAG
**Optimized 961**	TTCGCCACGTCCGACCGGCCGGTTCTGCTTAATAGCTGGGAAGGAGTTTATTTCGACTAC
**Original 961**	TTCGCCACGTCCGACCGGCCGGTTCTGCTGAATAGCTGGGAAGGTGTTTATTTCGACTAC
**Optimized 1021**	AATCAAAGCAGCATCGAAACTCTTGCCGAAGAGTCCGCTGCCCTGGGTGTCCACCTCTTT
**Original 1021**	AATCAAAGCAGCATCGAAACTCTTGCCGAAGAGTCCGCTGCCCTGGGTGTCCACCTCTTT
**Optimized 1081**	GTCATGGACGACGGCTGGTTTGGGGACAAGTACCCCCGAGTGTCCGATAACGCCGGACTG
**Original 1081**	GTCATGGACGACGGCTGGTTTGGGGACAAGTACCCTCGAGTGTCCGATAACGCCGGACTG
**Optimized 1141**	GGCGACTGGATGCCCAATCCAGCGCGCTTCCCGGACGGGTTGACCCCGGTCGTGCAAGAC
**Original 1141**	GGCGACTGGATGCCCAATCCAGCGCGCTTCCCGGACGGGTTGACCCCGGTCGTGCAAGAC
**Optimized 1201**	ATCACAAATCTCACCGTCAATGGCACAGAGTCCACAAAACTTCGCTTTGGTATTTGGGTG
**Original 1201**	ATCACAAATCTCACCGTCAATGGCACAGAGTCCACAAAACTTCGCTTTGGTATTTGGGTG
**Optimized 1261**	GAGCCCGAGATGGTCAACCCCAATTCCACTCTCTACCACGAACACCCGGAGTGGGCACTT
**Original 1261**	GAGCCCGAGATGGTCAACCCCAATTCCACTCTCTACCACGAACACCCGGAGTGGGCGCTT
**Optimized 1321**	CATGCCGGGCCTTACCCCCGTACCGAGCGTCGGAACCAGCTCGTCCTCAACCTGGCGCTT
**Original 1321**	CATGCCGGGCCTTACCCCCGTACCGAGCGTCGGAACCAGCTCGTCCTCAACCTGGCGCTT
**Optimized 1381**	CCGGCTGTGCAGGACTTCATCATAGACTTCATGACGAACCTGTTACAAGATACCGGCATT
**Original 1381**	CCGGCTGTGCAGGACTTCATCATAGACTTCATGACGAACCTGTTACAAGATACCGGCATT
**Optimized 1441**	TCCTACGTCAAATGGGACAACAACCGGGGGATACACGAGACGCCCTCTCCGTCCACTGAC
**Original 1441**	TCCTACGTCAAATGGGACAACAACCGGGGGATACACGAGACGCCCTCTCCGTCGACTGAC
**Optimized 1501**	CATCAGTACATGCTTGGCCTCTACCGGGTGTTCGACACACTGACCACCCGTTTTCCGGAT
**Original 1501**	CATCAGTACATGCTTGGCCTCTACCGGGTGTTCGACACACTGACCACCCGTTTTCCGGAT
**Optimized 1561**	GTCCTGTGGGAAGGATGTGCCTCGGGCGGTGGTAGATTTGATGCTGGCATGCTGCAGTAT
**Original 1561**	GTCCTGTGGGAAGGATGTGCCTCGGGCGGCGGCCGCTTTGATGCTGGCATGCTGCAGTAT
**Optimized 1621**	GTCCCCCAGATCTGGACTTCCGACAACACCGACGCCATCGACCGAATCACCATCCAATTT
**Original 1621**	GTCCCCCAGATCTGGACTTCCGACAACACCGACGCCATCGACCGAATCACCATCCAATTT
**Optimized 1681**	GGGACCTCGCTTGCCTACCCGCCATCAGCAATGGGAGCCCACCTCTCCGCGGTTCCTAAT
**Original 1681**	GGGACCTCGCTTGCCTACCCGCCATCAGCAATGGGAGCCCACCTCTCCGCGGTCCCTAAT
**Optimized 1741**	GCACAGACCGGTCGCACTGTGCCCTTTACTTTCCGCGCACACGTTGCTATGATGGGTGGT
**Original 1741**	GCACAGACCGGTCGCACTGTGCCCTTTACTTTCCGCGCACACGTTGCTATGATGGGTGGT
**Optimized 1801**	TCTTTCGGCTTGGAGTTGGACCCAGCGACGGTGGAAGGGGACGAAATAGTTCCCGAGTTG
**Original 1801**	TCTTTCGGCTTGGAGCTGGATCCGGCGACGGTGGAAGGGGACGAAATAGTTCCCGAGCTC
**Optimized 1861**	CTTGCGCTGGCGGAAAAAGTGAACCCTATCATTTTGAACGGAGATCTGTATCGGCTACGC
**Original 1861**	CTTGCGCTGGCGGAAAAGGTGAACCCTATCATTTTGAACGGAGATCTGTATCGGCTACGC
**Optimized 1921**	CTACCTCAAGACTCCCAGTGGCCTGCCGCACTCTTTGTGTCCCAGGATGGCGCACAGGCT
**Original 1921**	CTACCTCAAGACTCCCAGTGGCCTGCCGCACTCTTTGTGTCTCAGGATGGCGCACAGGCT
**Optimized 1981**	GTTCTGTTCTACTTCCAGGTCCAGCCGAATGTCAACCATGCCGTGCCGTGGGTCAGGCTG

**Original 1981**	GTTCTGTTCTACTTCCAGGTCCAGCCGAATGTCAACCATGCCGTGCCGTGGGTCAGGCTG
**Optimized 2041**	CAGGGGTTGGACCCTAAGGCGGACTATACCGTTGATGGAGATCAGACGTATTCCGGGGCA
**Original 2041**	CAGGGGTTGGACCCTAAGGCGGACTATACCGTCGATGGGGATCAGACGTATTCTGGAGCA
**Optimized 2101**	ACACTGATGAATCTGGGGTTGCAGTATAGTTTTGACACCGAGTATGGTAGCAAGGTAGTT
**Original 2101**	ACACTAATGAATCTGGGGTTGCAGTATAGTTTTGACACCGAGTATGGAAGCAAGGTAGTT
**Optimized 2161**	TTCCTGGAGAGGCAATGA
**Original 2161**	TTCCTGGAGAGGCAATGA

Figure A. Comparison of the original and optimized *aga* gene sequences. The optimized codons are shown in red.

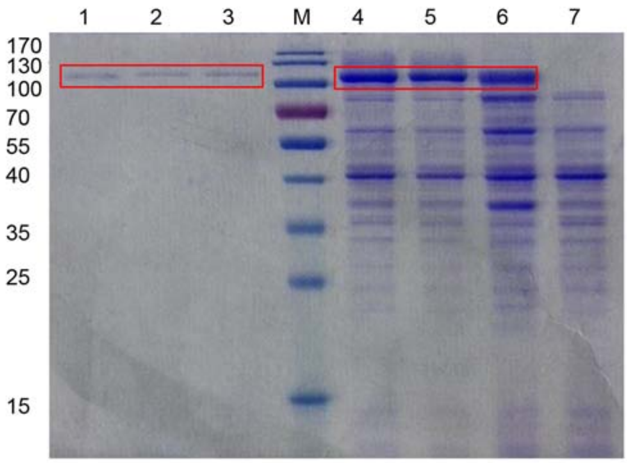

Figure B. SDS-PAGE (12%) analysis of the extracellular and intracellular proteins of AGA-X33, AGA-KM71H-2, AGA-KM71H-1. Induced for 144 h in shaking flask, 1: the extracellular proteins of AGA-KM71H-1; 2: the extracellular proteins of AGA-KM71H-2; 3: the extracellular proteins of AGA-X33; 4: the intracellular proteins of AGA-KM71H-1; 5: the intracellular proteins of AGA-KM71H-2; 6: the intracellular proteins of AGA-X33. 7: the control strain KM71H transformed with plasmid pPICZαA. The boxed bands are α-galactosidases (molecular mass: 115 kDa).

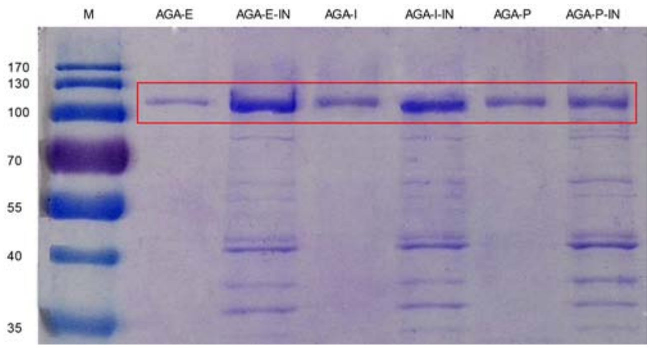

Figure C. SDS-PAGE (12%) analysis of the extracellular and intracellular proteins of AGA-E, AGA-I, and AGA-P (144 h post-induction in shaking flasks). AGA-E: the extracellular proteins of AGA-E; AGA-E-IN: the intracellular proteins of AGA-E; AGA-I: the extracellular proteins of AGA-I; AGA-I-IN: the intracellular proteins of AGA-I; AGA-P: the extracellular proteins of AGA-P; AGA-P-IN: the intracellular proteins of AGA-P. The boxed bands are α-galactosidases (molecular mass: 115 kDa).
