# Supplementary material for: Correction: Improving the Secretory Expression of an α-Galactosidase from Aspergillus niger in Pichia pastoris
Source: PLoS One. 2016 Oct 20;11(10):e0165186. doi: 10.1371/journal.pone.0165186 (PMC5072550; doi:10.1371/journal.pone.0165186)
Supplement: S2 File — (PDF) [file pone.0165186.s002.PDF]

RESEARCH ARTICLE

Improving the Secretory Expression of an α-Galactosidase from *Aspergillus niger* in *Pichia pastoris*

Xianliang Zheng[1,2,3], **Bo Fang**[1,2], **Dongfei Han**[1], **Wenxia Yang**[1], **Feifei Qi**[1], **Hui Chen**[1], **Shengying Li**[1]*

1 Shandong Provincial Key Laboratory of Synthetic Biology, and CAS Key Laboratory of Biofuels at Qingdao Institute of Bioenergy and Bioprocess Technology, Chinese Academy of Sciences, No. 189 Songling Road, Qingdao, Shandong 266101, China, **2** University of Chinese Academy of Sciences, Beijing 100049, China, **3** Sino-Danish Center for Education and Research, Beijing, 100190, China

* lishengying@qibebt.ac.cn

CrossMark
click for updates

OPEN ACCESS

Citation: Zheng X, Fang B, Han D, Yang W, Qi F, Chen H, et al. (2016) Improving the Secretory Expression of an α-Galactosidase from *Aspergillus niger* in *Pichia pastoris*. PLoS ONE 11(8): e0161529. doi:10.1371/journal.pone.0161529

Editor: Israel Silman, Weizmann Institute of Science, ISRAEL

Received: April 8, 2016

Accepted: August 8, 2016

Published: August 22, 2016

Copyright: © 2016 Zheng et al. This is an open access article distributed under the terms of the Creative Commons Attribution License, which permits unrestricted use, distribution, and reproduction in any medium, provided the original author and source are credited.

Data Availability Statement: All relevant data are within the paper and its Supporting Information files.

Funding: This work was financially supported by funding from the National Natural Science Foundation of China under grant numbers NSFC 21406250 (HC) and NSFC 31300663 (DH), the Applied Basic Research Programs of Science and Technology of Qingdao, 15-9-1-106-jch (HC), and the Opening Fund of Key Laboratory of Protein Chemistry and Developmental Biology of the Ministry of Education at Hunan Normal University, 2015DF05 (SL).

Abstract

α-Galactosidases are broadly used in feed, food, chemical, pulp, and pharmaceutical industries. However, there lacks a satisfactory microbial cell factory that is able to produce *α*-galactosidases efficiently and cost-effectively to date, which prevents these important enzymes from greater application. In this study, the secretory expression of an *Aspergillus niger α*-galactosidase (AGA) in *Pichia pastoris* was systematically investigated. Through codon optimization, signal peptide replacement, comparative selection of host strain, and saturation mutagenesis of the P1' residue of Kex2 protease cleavage site for efficient signal peptide removal, a mutant *P*. *pastoris* KM71H (Mut^s) strain of AGA-I with the specific P1' site substitution (Glu to Ile) demonstrated remarkable extracellular *α*-galactosidase activity of 1299 U/ml upon a 72 h methanol induction in 2.0 L fermenter. The engineered yeast strain AGA-I demonstrated approximately 12-fold higher extracellular activity compared to the initial *P*. *pastoris* strain. To the best of our knowledge, this represents the highest yield and productivity of a secreted *α*-galactosidase in *P*. *pastoris*, thus holding great potential for industrial application.

Introduction

As a subfamily of *exo*-glycosidases, *α*-galactosidases (D-galactoside galactohydrolase, EC. 3.2.1.22) are broadly distributed in microorganisms, plants and animals. These enzymes primarily catalyze the removal of *α*-1,6-linked terminal non-reducing galactose residues from various *α*-galactosides [1], such as melibiose, raffinose, stachyose, and others [2]. In addition, they also display transgalactosylation activity [3] and hemagglutination activity [4]. Because of these catalytic features, *α*-galactosidases have been extensively used in feed, food, chemical, pulp, and pharmaceutical industries [5].

Some *α*-galactosidases are important animal feed additives since they are able to enhance the nutritive quality and energy efficiency of feed by hydrolyzing non-metabolizable sugars in corns and legumes [6], thereby alleviating flatulence, diarrhea and anorexia of animals. The use of these enzymes in animal farming industries can also benefit environments by lowering the

Competing Interests: The authors have declared that no competing interests exist.

emission of carbon dioxide, methane and other greenhouse gases as well as incompletely digested fecal matter [7]. In pulp and paper industries, α-galactosidases are extensively utilized to improve the bleaching effect of mannanases on softwood Kraft pulp [8]. α-Galactosidases have also been developed into drugs, such as Fabrazyme^{TM}, to treat Fabry's disease [9]. Furthermore, some α-galactosidases have been reported to be applied for the conversion of erythrocyte antigens from Type B to Type O [5] and for xenotransplantation [10].

Currently, industrial α-galactosidases are mainly originated from filamentous fungi, such as *Aspergillus*, *Trichoderma* and *Penicillium* species [11], due to their extracellular localization, acidic pH stability, and thermostability [12]. To lower the cost for industrial production of α-galactosidases, many recombinant strains based on different heterologous hosts have been constructed. For example, a human α-galactosidase A was heterologously expressed in both *Escherichia coli* [13] and in the psychrophilic bacterium *Pseudoalteromonas haloplanktis* TAC125 isolated from Antarctic [13]. Although the full-length α-galactosidase could be produced in *E. coli*, the limited chaperone activity in this popular prokaryotic expression system only generated inactive proteins. In the psychrophilic expression system, the active full-length α-galactosidase was produced, albeit in low yield. Moreover, the low temperature required for active enzyme production was cost-prohibitive for industrial manufacturing [13].

Yeast expression systems such as *Saccharomyces cerevisiae* [14] and *Pichia pastoris* [15] were also employed for α-galactosidase production. For instance, the α-galactosidase genes from *Bispora* sp. MEY-1, *Penicillium* sp. F63, and *Rhizomucor miehei* were expressed in *P. pastoris*. Their extracellular enzymatic activities, as determined with the PNPG (*p*-nitrophenyl-α-D-galactopyranoside) method [16], were reported to be 1.5 U/ml [17], 111 U/ml [18] and 240 U/ml [19], respectively, upon a 96 h methanol induction. When an α-galactosidase gene from *Penicillium janczewskii zalesk* was mutated by error-prone PCR and DNA shuffling, and then expressed in *P. pastoris* X33 [20], the extracellular α-galactosidase activity reached 420 U/ml during a 72 h post-induction phase in 10 L fermenter.

To enhance heterologous protein expression in *P. pastoris*, various approaches have been explored, such as screening of different methanol induction intensities [21], overexpression of molecular chaperones for better protein folding and secretion [22], and optimization of the secretory signal peptide [23]. However, these strategies have not yet been applied for improvement of the secretory expression of α-galactosidase in *P. pastoris*. In the present work, therefore, we systematically applied codon optimization, signal peptide replacement, comparative selection of host strains, and saturation mutagenesis of the P1' residue of Kex2 protease cleavage site to an α-galactosidase from *Aspergillus niger* (AGA) in order to engineer a *P. pastoris* cell factory for cost-effective production of AGA. As expected, the best mutant strain AGA-I with the specific P1' site substitution (Glu to Ile) achieved a significant extracellular α-galactosidase activity of 1299 U/ml upon a 72 h methanol induction in 2.0 L fermenter. To the best of our knowledge, this represents the highest reported secretory expression level of α-galactosidase so far.

Materials and Methods

Plasmid, strains, media and reagents

The plasmid pPICZαA and the yeast strain *P. pastoris* X-33 were purchased from Invitrogen (San Diego, CA, US). The *P. pastoris* KM71H (Invitrogen, San Diego, CA, US) was a gift from Professor Jing Wu of Jiangnan University. *A. niger* (Taxonomy ID: 425011) and *E. coli* DH5α were reserved in our laboratory. All used restriction enzymes and genetic manipulation kits were supplied by Takara (Dalian, China). The antibiotic zeocin was bought from Solarbio (Beijing, China). Yeast nitrogen base (YNB), yeast extract, tryptone and biotin were from Oxoid (Beijing, China). All organic solvents and reagents used in this study were analytical grade.

Recipes for used media were as follows: YPDS medium: 2% tryptone, 1% yeast extract, 1% dextrose, 1 M sorbitol, 1.5% agar, supplemented with certain concentrations of zeocin. BMGY medium: 2% tryptone, 1% yeast extract, 100 mM potassium phosphate buffer, pH 7.0, 1.34% YNB, 0.4 ppm biotin, and 1% (v/v) glycerol. BMMY medium: 2% tryptone, 1% yeast extract, 100 mM potassium phosphate buffer, pH 7.0, 1.34% YNB, 0.4 ppm biotin, and 1% (v/v) methanol. Low salt LB medium: 1% tryptone, 0.5% yeast extract, 0.5% NaCl, supplemented with 25 μg/ml zeocin when required, and 1.5% agar powder was added for agar plate preparation. Basal salt medium: 4% glycerol, 1.65% K_2SO_4, 1.35% $MgSO_4$, 1.5% $NH_4H_2PO_4$, 0.46% KH_2PO_4, 0.09% $CaSO_4$, 0.136% KOH, and 12 ml/l PTM1. PTM1: 0.6% $CuSO_4{\cdot}5H_2O$, 0.008% NaI, 0.3% $MnSO_4{\cdot}H_2O$, 0.002% H_3BO_3, 0.02% $Na_2MoO_4{\cdot}2H_2O$, 0.05% $CoCl_2$, 2% $ZnCl_2$, 6.5% $FeSO_4{\cdot}7H_2O$, 0.5% H_2SO_4 (95%), and 0.02% biotin.

Construction of plasmids and recombinant strains

The *A*. *niger* α-galactosidase encoding gene *aga* (Genbank Accession No. AJ251873.1) was codon-optimized for *P*. *pastoris* (Fig A in S1 File) and synthesized by GenScript (Nanjing, China). Using the synthetic gene as template, the *aga* fragment, without the native signal peptide, was amplified by PCR with primers as follows: *forward*, 5'-CCGGAATTCGCACCCGCA ATTGGG-3' (the underlined nucleotides indicate the *Eco*RI restriction site); *reverse*, 5'-ATAG CGGCCGCTCATTGCCTCTCCA-3' (the underlined bases denote the *Not*I cutting site). Next, the *Eco*RI and *Not*I doubly digested *aga* fragment was ligated into the *Eco*RI/*Not*I pretreated plasmid pPICZαA. The resulted plasmid was transformed into *E*. *coli* DH5α competent cells. A selected transformant grown on low salt LB medium containing 25 μg/ml zeocin was picked for preparation of pPICZαA-*aga*, whose sequence was confirmed by DNA sequencing at Sangon, Shanghai.

For the site-specific mutagenesis of the Kex2 P1' site, 19 pairs of mutagenic overlapping primers (Table A in S1 File) were designed, and synthesized by Sangon, Shanghai. With each pair of primers, pPICZαA-*aga* was amplified using the high-fidelity *Pfu* DNA polymerase (Thermo Fisher Scientific, USA) to afford 19 mutated pPICZαAX-*aga* plasmids, each containing a different amino acid codon at the Kex2 P1' site, respectively. The wild-type PCR template was removed by *Dpn*I digestion, and the mutated pPICZαAX-*aga* was transformed into *E*. *coli* DH5α competent cells.

For yeast transformation, pPICZαA-*aga* or an individual pPICZαAX-*aga* was linearized by *Sac*I and transformed into competent cells of *P*. *pastoris* X-33 or *P*. *pastoris* KM71H via electroporation. The transformation conditions were as follows: 0.2 cm cuvette, 1500 V, 200 Ω, 25 μF. YPDS plates containing 100 μg/ml zeocin were used for preliminary selection of the positive transformants harboring chromosomal integration of *aga* expression cassettes. YPDS plates supplemented with gradually increasing concentration of zeocin (500–2000 μg/ml) were used for further screening of multi-copy integrated recombinants.

Yeast expression of the recombinant AGA

A single colony of *P*. *pastoris* containing multi-copy AGA expression cassettes was grown in 2 ml YPD medium in a rotary shaker at 30°C, 250 rpm for 24 h. 20 μl seed culture was inoculated to 25 ml BMGY medium in a 250 ml baffled flask and grown until the OD_{600} of culture reached 4.0–6.0. Then, yeast cells were harvested by centrifugation at 5,000 *g* for 5 min and re-suspended in 100 ml fresh BMMY medium in a 500 ml baffled flask. The cells were further grown in a rotary shaker at 30°C, 250 rpm. Next, 1% (v/v) methanol was added into culture every 24 h for induction of α-galactosidase expression. The strain harboring empty vector pPICZαA was subject to the same manipulation as control for expression analysis.

Determination of cell density and enzymatic activity

At designated time points, 1 ml aliquots of induced cell culture in BMMY medium were withdrawn for determination of cell density by measuring OD_{600}. The cultures were then centrifuged at 5,000 *g* for 10 min, and the supernatants were used as testing samples for measurement of extracellular enzymatic activity. The intracellular enzyme was prepared by following Manual of EasySelect *Pichia* Expression Kit (Invitrogen). Briefly, the frozen-thawed cell pellets were placed on ice. To each cell pellet sample from 1 ml culture, 100 μl Breaking Buffer (50 mM sodium phosphate, pH 7.4, 1 mM PMSF, 1 mM EDTA, 5% glycerol) was added to resuspend cells. Next, an equal volume of acid-washed glass beads (Φ 0.5 mm, Sigma Aldrich) was added into cell suspension. The mixture was vortexed for 30 sec, incubated on ice for 30 sec, and repeated for 8 cycles. After centrifugation at 16,000 *g* for 10 min at 4°C, the clear supernatant containing intracellular enzymes was transferred to a clean microcentrifuge tube. Breaking Buffer was added to make the volume up to 1 ml, and the final 1 ml solution was used for enzymatic activity measurement.

The enzymatic assay for AGA activity [16] was carried out in the reaction mixture containing 2 mM PNPG (*p*-nitrophenyl-*α*-D-galactopyranoside) as substrate, and 200 μl diluted enzyme solution in 1 ml of 0.05 M acetic buffer (pH 5.5). The AGA catalyzed reactions were carried out at 40°C for 10 min and was quenched by adding 1 ml of 0.5 M sodium carbonate solution. The released *p*-nitrophenyl was quantified by measuring the absorbance at 405 nm. One unit of enzyme activity was defined as the amount of enzyme that releases 1.0 μmol of *p*-nitrophenyl per min at pH 5.5 and 40°C. The unit of enzymatic activity is indicated as U/ml, where ml represents 1 ml of cleared culture (with cells removed) for the secreted activity, or, 1 ml of Breaking buffer containing the cell lysate from 1 ml of culture for the intracellular activity. All AGA activities reported in this study were determined using the PNPG method.

High-density fermentation

The high-density fermentation of the unmutated strain AGA-E and the two selected mutant strains of AGA-P and AGA-I was performed in 2.0 L fermentation tanks (New Brunswick USA). The process of high-density fermentation had three stages including the cell growth stage, the supplementary food culture stage, and the induced enzyme production stage. The cultivation was maintained at constant pH using ammonium hydroxide. The ventilation rate was 1:1.2, and the dissolved oxygen was controlled between 20–30% during the whole process through adjustment of agitation and airflow. Fermentations were performed by following the *Pichia* Fermentation Guidelines (Version B, 053002, Invitrogen Inc.) with some modifications. First, the seed culture was inoculated to the basal salt medium (containing 12 ml/l of PTM1 trace elements solution) with 10% inoculation ratio in a 2.0 L fermenter with a 1.0 L working volume. The cultivation temperature was set to 29°C, and the pH was maintained at 5.0. Second, when glycerol was used up, 200 ml 50% glycerol was supplemented with the speed of 16–25 ml/l/h. Finally, when the OD_{600} of culture reached ~250 between 28–32 h, the supplementation of glycerol was stopped to starve the cells for 2–3 h, followed by flowing methanol to initiate the induced enzyme production stage. The flow rate of methanol was improved from 1.3 ml/l/h to 6 ml/l/h gradually. Samples were taken at certain time points to monitor OD_{600} and AGA activity.

GenBank accession numbers

The DNA sequence of the codon-optimized *A. niger* *α*-galactosidase encoding gene *aga* and the mutated AGA-I gene were submitted to GenBank with the assigned accession numbers of KU695596 and KU695597, respectively.

Results

Expression of AGA in *P. pastoris* X33 and KM71H

To select an appropriate yeast strain for secretory expression of the α-galactosidase from *A. niger* (AGA), the recombinant plasmid pPICZαA-*aga* was transformed to *P. pastoris* X33 (Mut^+, the methanol utilization plus phenotype) and *P. pastoris* KM71H (Mut^s, the methanol utilization slow phenotype), respectively. Through homologous recombination between the recombinant vector and the yeast genome, multiple chromosomal integration of the expression cassette consisting of the *aga* gene with its native signal peptide sequence replaced by the α-factor signal peptide sequence from *S. cerevisiae*, and a selectable zeocin resistance gene (*zeo*^{r}), onto the AOX1 (alcohol oxidase 1) locus of yeast genome were achieved. The multiple single crossover events could occur at the 5' AOX1 promoter region or the 3' AOX1 terminator region [24]. YPDS plates supplemented with gradually increased concentration (500–2000 μg/ml) of zeocin were used to screen positive transformants that harbor more copies of integrated AGA expression cassettes.

After several rounds of high concentration antibiotic screening, one positive recombinant *P. pastoris* X33 strain (AGA-X33) and two positive recombinant strains of *P. pastoris* KM71H (AGA-KM71H-1 and AGA-KM71H-2) containing multi-copy AGA gene cassettes, as demonstrated by good resistance to 2000 μg/ml of zeocin, were selected for fermentation of AGA in shaking flasks. A two-stage fermentation was adopted for expressing AGA: 1) At the cell growth stage, biomass was accumulated in BMGY medium until the cell density reached OD_{600} between 4.0 and 6.0. This step was aimed to avoid the potential toxicity caused by the expression inducer methanol; 2) At the inducible expression stage, the AGA expression was induced in BMMY medium by feeding methanol, which acts as both protein expression inducer and carbon source for further biomass production.

During shaking flask fermentation of AGA-X33, AGA-KM71H-1 and AGA-KM71H-2 for 168 h (144 post-induction hours), we monitored the OD_{600} of these three strains. Expectedly, AGA-KM71H-1 and AGA-KM71H-2 (Mut^s, the methanol utilization slow phenotype) grew significantly slower than AGA-X33 (Mut^s, the methanol utilization plus phenotype) (Fig 1A). The AGA activity of the fermentation broth and the intracellular portion were determined with the PNPG method [16]. As results, the extracellular AGA activity of AGA-X33 was determined to be 160 U/ml, which was lower than that of AGA-KM71H-1 (248 U/ml) or AGA-KM71H-2 (236 U/ml) (Fig 1B). Meanwhile, the intracellular AGA activity of AGA-X33 was determined to be 1988 U/ml, which was higher than that of AGA-KM71H-1 (1467 U/ml) or AGA-KM71H-2 (1358 U/ml) (Fig 1B). These results are consistent with the AGA protein quantity reflected by SDS-PAGE analysis (Fig B in S1 File). Apparently, most AGA produced by AGA-X33 (Mut^+) was not secreted into the extracellular space. When *P. pastoris* KM71H (Mut^s) was used, the secretory yield of AGA was improved for 56% while the intracellular AGA activity decreased compared to that in *P. pastoris* X33. Therefore, *P. pastoris* KM71H was selected as the heterologous host strain for secretory expression of AGA in the following study.

Saturation mutagenesis of the Kex2 P1' site for improving the secretory expression of AGA

Although the recombinant *P. pastoris* KM71H strain displayed enhanced secretory ability for AGA, the intracellular AGA activity (and amount) still accounted for the majority of total AGA activity (and amount) (Fig 1B and Fig B in S1 File), indicating that the secretion of AGA in *P. pastoris* KM71H still needs to be improved. It has been known that protein secretion in *P.*

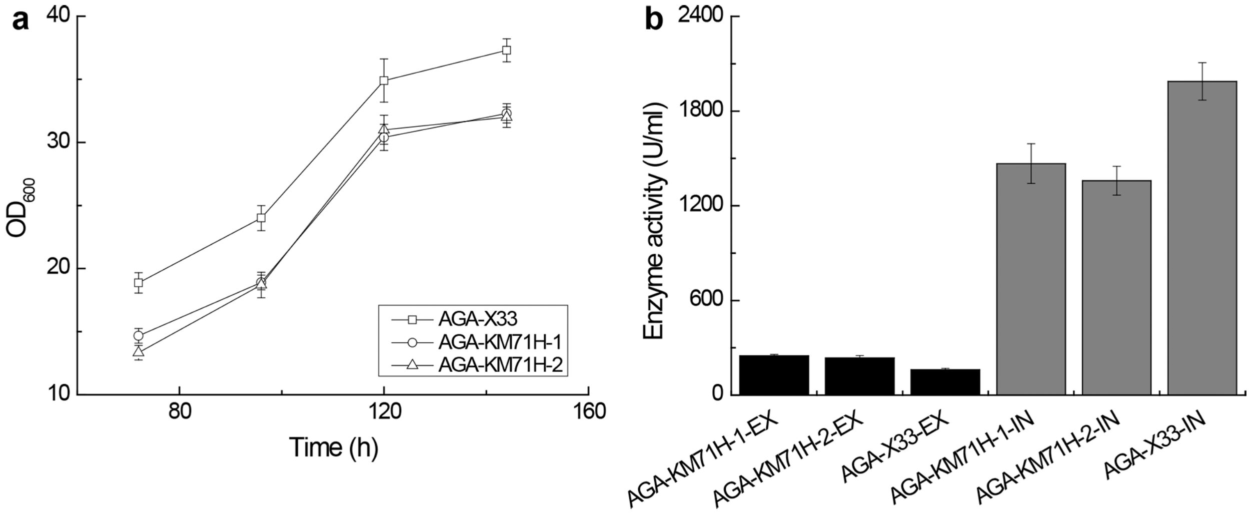

Fig 1. (a) Growth curves of AGA-X33, AGA-KM71H-1 and AGA-KM71H-2. (b) The extracellular AGA activity and the intracellular AGA activity of the recombinant *P. pastoris* strains. Including AGA-KM71H-1, AGA-KM71H-2, and AGA-X33 (induced for 144 h in shaking flask). AGA-KM71H-1, AGA-KM71H-2: the two selected strains with AGA expressed in *P. pastoris* KM71H; AGA-X33: the strain with AGA expressed in *P. pastoris* X33. EX: the extracellular AGA activity; IN: the intracellular AGA activity. All measurements were performed with three parallel samples.

doi:10.1371/journal.pone.0161529.g001

pastoris follows the pathway of endoplasmic reticulum→Golgi complex→vesicle→extracellular space. During this post-translational translocation process, one of the most important steps is the site-specific cleavage of signal peptide from pre-protein [25].

In this work, we took advantage of the *α*-factor signal peptide to guide the AGA secretion in *P. pastoris*. This 85 amino acid *α*-factor signal peptide originally from *S. cerevisiae* represents one of the most frequently used signal peptides in yeast expression vectors [26]. As designed, this signal peptide can be cut off by the yeast endoprotease Kex2 [27]. The recognition and cutting sites of Kex2 including P1, P2, and P1' [28] are shown in Fig 2A. The P1 position is well conserved with an arginine residue for Kex2 recognition. The P2 site is the second basic amino acid site, where either arginine or lysine can be recognized by Kex2 [29]. According to previous studies, differential amino acid residues at the P1' site could significantly affect the cutting efficiency by Kex2 and hence the final amount of secretory proteins [21, 30].

Thus, saturation mutagenesis was elected to optimize the amino acid residue at the P1' site of Kex2 in order to find out the best option for the secretory expression of *α*-galactosidase. Upon generation of a mutant library containing 19 differently substituted P1' site, *P. pastoris* KM71H was transformed with each of these mutant expression vectors. Comparatively, the highest secretory expression level was achieved by two mutant strains AGA-I and AGA-P (*i.e.* isoleucine and proline at the P1' site, respectively). The extracellular AGA activity of AGA-I and AGA-P turned out to be 549 U/ml and 560 U/ml respectively, both of which showed approximately 70% improvement compared to that of the unmutated strain AGA-E (323 U/ml) (Fig 2B). Notably, AGA-P increased the secretory expression of AGA at the cost of decreased intracellular expression (840 U/ml), whereas AGA-I improved the extracellular and intracellular expression (2,613 U/ml) at the same time (Fig 2C). Again, these results are consistent with the AGA protein quantity reflected by SDS-PAGE analysis (Fig C in S1 File). Interestingly, AGA-V (Glu to Val) completely lost the activity to produce *α*-galactosidase (Fig 2B and 2C) for unknown reasons.

To confirm that the increased AGA activity of P1' mutants was due to more efficient secretion other than modified *N*-terminus, the extracellular and intracellular AGAs produced by

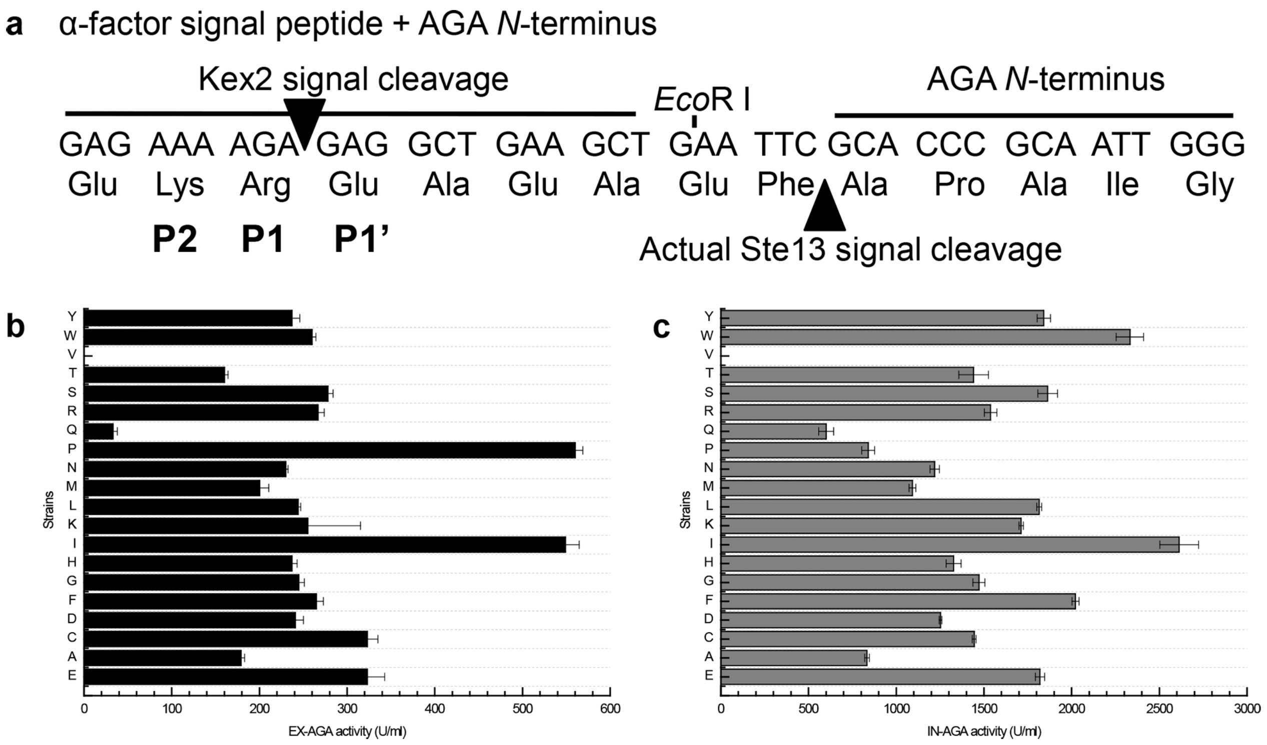

Fig 2. (a) The Kex2 signal peptide cleavage site. The extracellular (b) and intracellular (c) AGA activity of the *P. pastoris* strains (KM71H) carrying alternative Kex2 P1' residues. Induced for 144h in shaking flask, E: the unmutated strain carrying Glu at the P1' site. Mutant strains are presented by the substituting amino acid at the P1' site in abbreviation. All measurements were performed in triplicate.

doi:10.1371/journal.pone.0161529.g002

both AGA-E (the unmutated strain) and the AGA-P mutant strain were submitted to *N*-terminal protein sequencing by Edman degradation [31] at Chinese Academy of Agricultural Sciences. The starting five amino acids of all four samples were unanimously A-P-A-I-G, which results from sequential cleavages of the premature enzyme by Kex2 and Ste13 (Fig 2A). These results clearly indicate that the P1' site mutation affected the secretory efficiency rather than AGA protein sequence.

High-density fermentation of AGA-I and AGA-P

The secretory expression of AGA by AGA-I and AGA-P was significantly improved in shaking flask fermentation. During their (and other mutants') fermentation processes, we noticed that the pH value could drop to 2.0 after induction for 96 h (data not shown). This strong acidic condition significantly hindered the growth of cells and likely in turn deteriorated enzyme production and secretion. To solve this problem and to provide *P. pastoris* with a better growth environment for AGA production, the controlled high-density fermentation in fermentation tank was performed.

Experimentally, the two mutants AGA-I and AGA-P were cultured in 2.0 L fermentation tanks, and the unmutated strain AGA-E was fermented in parallel as control. Under the conditions of 20–30% dissolved oxygen and steady pH value of 5.0, three strains reached the OD_{600} values between 320–340 (Fig 3A) after 144 h methanol induction. Regarding the AGA secretion capacity, AGA-I and AGA-P showed the highest extracellular activity of 1370 U/ml and 1339 U/ml, respectively. Longer fermentations gave decreased α-galactosidase activity (data not shown). In the meantime, we measured the extracellular total protein concentration

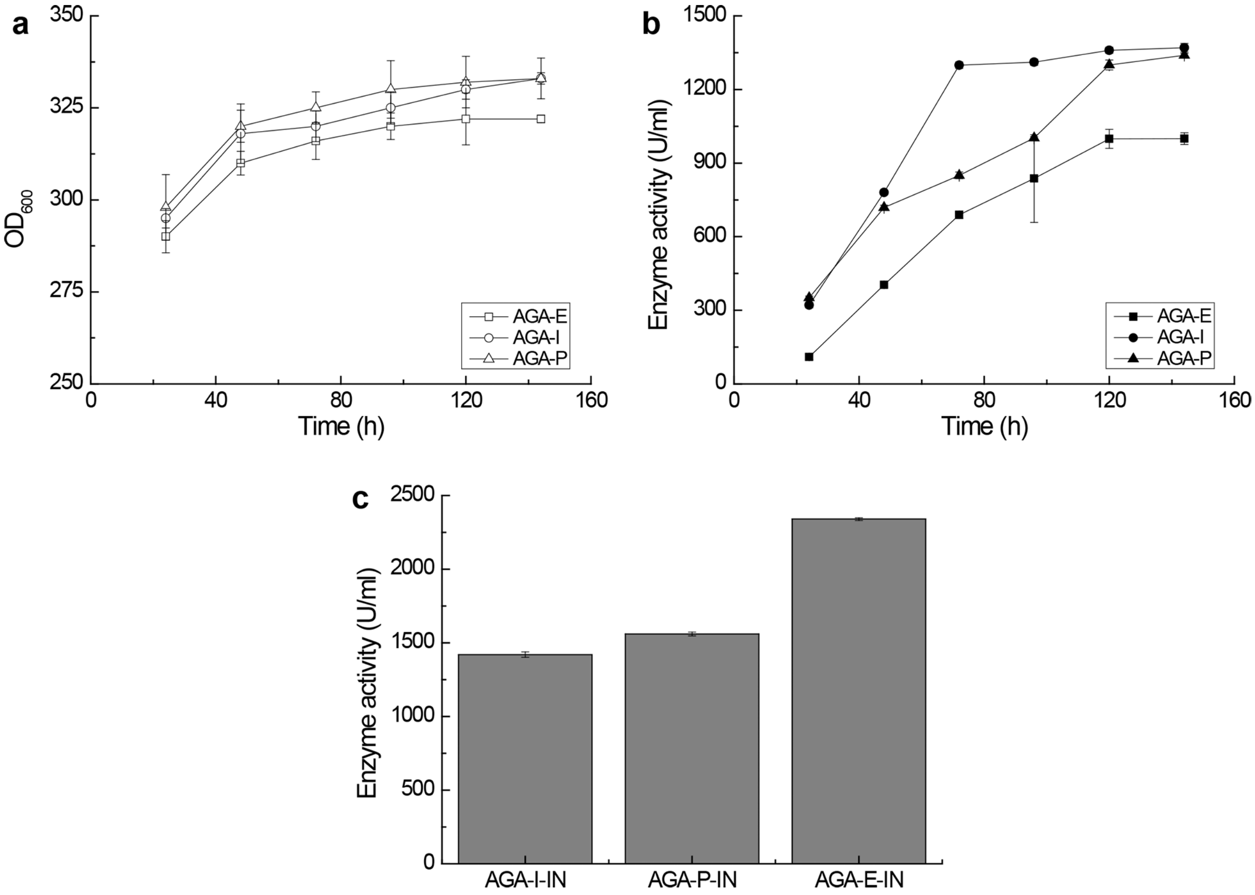

Fig 3. (a) Growth curves of the three selected strains in 2.0 L fermentation tank. (b) The time course of the extracellular AGA activity of the three selected strains. (c) The intracellular enzymatic activity of AGA-I, AGA-P, and AGA-E. AGA-E: the strain with the unmutated P1' site; AGA-I and AGA-P: the two mutant strains with the P1' residue replaced by Ile and Pro, respectively. The intracellular activity of AGA-I, AGA-P and AGA-E were determined after 144 h induction in 2.0 L fermentation tanks. All measurements were performed in triplicate.

doi:10.1371/journal.pone.0161529.g003

(ETPC) of these samples to reflect the secretory expression level of AGAs in different strains because *P*. *pastoris* normally secrets very low levels of endogenous proteins [32] and AGAs accounted for > 90% of total proteins in media (Figs B and C in S1 File). The ETPC of AGA-I and AGA-P were determined to be 622 mg/l and 576 mg/l, respectively. Of particular importance, the extracellular activity of AGA-I reached 1299 U/ml (95% of the highest activity, ETPC: 533 mg/l) after 72 h induction, while the corresponding activity of AGA-P and AGA-E were only 850 U/ml (ETPC: 368 mg/l) and 689 U/ml (ETPC: 264 mg/l), respectively (Fig 3B). This is a fairly important result since the significantly shortened fermentation time would provide higher productivity and hence much lower costs. Moreover, the intracellular AGA activity of AGA-I and AGA-P were 1420 U/ml and 1560 U/ml (Fig 3C) after 144 h induction, respectively, both of which were lower than that of the unmutated strain AGA-E (2340 U/ml).

Discussion

In this work, AGA was first overexpressed in *P*. *pastoris* X33 (Mut^+). It turned out that the intracellular AGA activity was much higher than the extracellular AGA activity (Fig 1B). We hypothesized that AGA might be produced too fast to be efficiently processed by the secretory

machinery of *P. pastoris* X33. To test this hypothesis, AGA was then expressed in *P. pastoris* KM71H (Mut^s). Unlike *P. pastoris* X33 (Mut^+) that has two alcohol oxidase encoding genes (AOX1 and AOX2), the Mut^s strain *P. pastoris* KM71H only has AOX2 gene. Since the majority of alcohol oxidase activity in yeast is attributed to AOX1 gene product and AOX2 is a weaker alcohol oxidase than AOX1 [33], a Mut^s strain grows much slower than a Mut^+ strain when using methanol as major carbon source [34]. By lowering the growth rate and hence the protein expression rate, *P. pastoris* KM71H could be a better producer for foreign secretory proteins than a Mut^+ strain [32, 35, 36]. As expected, although both AGA-KM71H-1 and AGA-KM71H-2 grew slower than AGA-X33 (Fig 1A), they showed improved secretory expression of AGA, while the intracellular AGA activity decreased compared to *P. pastoris* X33. Additionally, a Mut^s strain does not require large amounts of methanol routinely used for large-scale fermentation of a Mut^+ strain. This represents another important advantage for practical application.

The cleavage of signal peptide from pre-protein significantly affects the efficiency of secretory pathway [25]. In this work we selected α-factor as the secretory guider for AGA. This 85-amino acid signal peptide can be processed by yeast endoprotease Kex2 [26]. The recognition and cutting sites of Kex2 includes P1, P2, and P1' sites [28]. The P1 position is well conserved with an arginine residue for Kex2 recognition. The P2 site is the second basic amino acid site, where either arginine or lysine can be recognized by Kex2 [29]. The P1' residue following the precursor dibasic processing sites likely play an important role of anchoring the recognition motif to the catalytic site of processing endoprotease [30], it may also affect the efficiency of further cleavage by Ste13 protease [37].

Our saturation mutagenesis of the P1' residue identified two optimal substitutions including E→I and E→P. Interestingly, Yang *et al.* reported that the best P1' site residues for secretory production of Venus protein and luciferase in *P. pastoris* were serine and asparagine, respectively [21]. Another previous study demonstrated that substituting the P1' residue of various prohormones or peptide precursors with the β-carbon branched side chain residues (Thr, Val, Leu, Ile), or Pro, Cys, Met and Trp were unfavorable for Kex2 cleavage [30]. Together with our results (Fig 2), it is evident that an optimal P1' site for the best Kex2 cutting efficiency is dependent on the target protein to be secreted. There is not a consensus residue at this protease recognition site. Thus, saturation mutagenesis of the P1' residue is recommended to be conducted in order to find out the best combination.

In consideration of the significant intracellular activity of AGA-I accounting for approximately one half of total activity (Fig 3B and 3C), more research is required for further improvement of AGA secretion. For example, overexpression of molecular chaperones such as HAC1, Bip/Kar2, or Pdi [22] and optimization of the fermentation process may further improve the protein secretory pathway of AGA, which is currently ongoing in our laboratory.

Conclusions

Through codon optimization, signal peptide replacement, comparative selection of host strain, and saturation mutagenesis of the P1' residue of Kex2 protease cleavage site, a novel recombinant *P. pastoris* strain AGA-I was developed for improved secretory production of an *A. niger* α-galactosidase. Compared to the initial *P. pastoris* X33 strain, this engineered strain demonstrated approximately 12-time higher extracellular activity (1299 U/ml *vs* 110 U/ml) after a 72 h methanol induction. To the best of our knowledge, this represents the highest yield and productivity of a secreted α-galactosidase in *P. pastoris*. It holds great potential for industrial application and is of great value in reducing energy consumption and production costs during fermentation process.

Supporting Information

S1 File. Sequences of the primers for constructing the mutant library (Table A); Comparison of the original and optimized *aga* gene sequences (Fig A); SDS-PAGE (12%) analysis of the extracellular and intracellular proteins of AGA-X33, AGA-KM71H-2, AGA-KM71H-1 (Fig B); and SDS-PAGE (12%) analysis of the extracellular and intracellular proteins of AGA-E, AGA-I, and AGA-P (144 h post-induction in shaking flasks) (Fig C).
(PDF)

Acknowledgments

We thank Professor Jing Wu of Jiangnan University for sharing the strain of *P*. *pastoris* KM71H. We are grateful to Dr. Huifang Xu (Qingdao Institute of Bioenergy and Bioprocess Technology, Chinese Academy of Sciences) for proofreading the manuscript.

Author Contributions

Conceptualization: XZ SL.

Formal analysis: XZ DH SL.

Funding acquisition: DH HC SL.

Investigation: XZ BF DH WY FQ HC.

Methodology: XZ HC SL.

Project administration: DH HC SL.

Resources: SL.

Supervision: SL.

Validation: XZ SL.

Writing - original draft: XZ.

Writing - review & editing: XZ SL.

References

1. Viana PA, de Rezende ST, Marques VM, Trevizano LM, Passos FML, Oliveira MGA, et al. Extracellular alpha-galactosidase from *Debaryomyces hansenii* UFV-1 and its use in the hydrolysis of raffinose oligosaccharides. J Agr Food Chem. 2006; 54(6):2385–91. doi: 10.1021/jf0526442 PMID: WOS:000236227100062.
2. Jaekoo Lee I, Cho Jaiesoon. Production and partial characterization of α-galactosidase activity from an Antarctic bacterial isolate, *Bacillus* sp. LX-1. Afr J Biotechnol. 2012; 11(60):12396–405.
3. Kato K IT, Kono H, Yamauchi R, Ueno Y. Transferase action of alpha-galactosidase from tubers of *Stachys affinisi*. Agric Biol Chem. 1982; 46:1089–90.
4. Delcampillo E, Shannon LM. An Alpha-Galactosidase with Hemagglutinin Properties from Soybean Seeds. Plant Physiol. 1982; 69(3):628–31. doi: 10.1104/Pp.69.3.628 PMID: ISI:A1982NG22500016.
5. Zhang YP, Gong F, Bao GQ, Gao HW, Ji SP, Tan YX, et al. B to O erythrocyte conversion by the recombinant alpha-galactosidase. Chinese Med J-Peking. 2007; 120(13):1145–50. PMID: ISI:000248102200006.
6. Ghazi S, Rooke JA, Galbraith H. Improvement of the nutritive value of soybean meal by protease and alpha-galactosidase treatment in broiler cockerels and broiler chicks. Brit Poultry Sci. 2003; 44(3):410–8. doi: 10.1080/00071660310001598283 PMID: ISI:000184576400012.
7. Patil AGG, Kumar SKP, Mulimani VH, Veeranagouda Y, Lee K. alpha-Galactosidase from *Bacillus megaterium* VHM1 and Its Application in Removal of Flatulence-Causing Factors from Soymilk. J

Microbiol Biotechn. 2010; 20(11):1546–54. doi: 10.4014/jmb.0912.12012 PMID: ISI:000284866800013.

8. Puchart V, Vrsanska M, Bhat MK, Biely P. Purification and characterization of alpha-galactosidase from a thermophilic fungus *Thermomyces lanuginosus*. Bba-Gen Subjects. 2000; 1524(1):27–37. doi: 10.1016/S0304-4165(00)00138-0 PMID: ISI:000165457900004.
9. Tsuboi K, Yamamoto H. Clinical observation of patients with Fabry disease after switching from agalsidase beta (Fabrazyme) to agalsidase alfa (Replagal). Genet Med. 2012; 14(9):779–86. doi: 10.1038/Gim.2012.39 PMID: ISI:000308767600003.
10. Zeyland J, Gawronska B, Juzwa W, Jura J, Nowak A, Slomski R, et al. Transgenic pigs designed to express human alpha-galactosidase to avoid humoral xenograft rejection. J Appl Genet. 2013; 54 (3):293–303. doi: 10.1007/s13353-013-0156-y PMID: ISI:000322188600006.
11. Manzanares P, de Graaff LH, Visser J. Characterization of galactosidases from *Aspergillus niger*: purification of a novel alpha-galactosidase activity. Enzyme Microb Tech. 1998; 22(5):383–90. doi: 10.1016/S0141-0229(97)00207-X PMID: WOS:000072918300013.
12. Aleksieva P, Tchorbanov B, Nacheva L. High-Yield Production of Alpha-Galactosidase Excreted from *Penicillium Chrysogenum* and *Aspergillus Niger*. Biotechnol Biotec Eq. 2010; 24(1):1620–3. doi: 10.2478/V10133-010-0015-5 PMID: WOS:000276230500015.
13. Unzueta U, Vazquez F, Accardi G, Mendoza R, Toledo-Rubio V, Giuliani M, et al. Strategies for the production of difficult-to-express full-length eukaryotic proteins using microbial cell factories: production of human alpha-galactosidase A. Appl Microbiol Biot. 2015; 99(14):5863–74. doi: 10.1007/s00253-014-6328-9 PMID: WOS:000356806300010.
14. Shibuya H, Nagasaki H, Kaneko S, Yoshida S, Park GG, Kusakabe I, et al. Cloning and high-level expression of alpha-galactosidase cDNA from *Penicillium purpurogenum*. Appl Environ Microbiol. 1998; 64(11):4489–94. Epub 1998/10/31. PMID: 9797312; PubMed Central PMCID: PMCPmc106674.
15. Chen Y, Jin M, Egborge T, Coppola G, Andre J, Calhoun DH. Expression and characterization of glycosylated and catalytically active recombinant human alpha-galactosidase A produced in *Pichia pastoris*. Protein expression and purification. 2000; 20(3):472–84. Epub 2000/11/23. doi: 10.1006/prep.2000.1325 PMID: 11087687.
16. Denherder IF, Rosell AMM, Vanzuilen CM, Punt PJ, Vandenhondel AMJJ. Cloning and Expression of a Member of the *Aspergillus-Niger* Gene Family Encoding Alpha-Galactosidase. Mol Gen Genet. 1992; 233(3):404–10. PMID: WOS:A1992JB37200010.
17. Wang H, Luo HY, Li JA, Bai YG, Huang HG, Shi PJ, et al. An alpha-galactosidase from an *acidophilic Bispora* sp MEY-1 strain acts synergistically with beta-mannanase. Bioresource Technol. 2010; 101 (21):8376–82. doi: 10.1016/j.biortech.2010.06.045 PMID: WOS:000280570900052.
18. Mi SJ, Meng K, Wang YR, Bai YG, Yuan TZ, Luo HY, et al. Molecular cloning and characterization of a novel alpha-galactosidase gene from *Penicillium* sp F63 CGMCC 1669 and expression in *Pichia pastoris*. Enzyme Microb Tech. 2007; 40(5):1373–80. doi: 10.1016/j.enzmictec.2006.10.017 PMID: WOS:000245519000055.
19. Chen Z, Yan QJ, Jiang ZQ, Liu Y, Li YC. High-level expression of a novel alpha-galactosidase gene from *Rhizomucor miehei* in *Pichia pastoris* and characterization of the recombinant enyzme. Protein expression and purification. 2015; 110:107–14. doi: 10.1016/j.pep.2015.02.015 PMID: WOS:000353313500016.
20. Chen YQ, Zhang B, Pei HL, Lv JN, Yang WH, Cao YH, et al. Directed Evolution of *Penicillium janczewskii zalesk* alpha-Galactosidase Toward Enhanced Activity and Expression in *Pichia pastoris*. Appl Biochem Biotech. 2012; 168(3):638–50. doi: 10.1007/s12010-012-9806-5 PMID: WOS:000309869100014.
21. Yang S, Kuang Y, Li HB, Liu YH, Hui XY, Li P, et al. Enhanced Production of Recombinant Secretory Proteins in *Pichia pastoris* by Optimizing Kex2 P1' site. Plos One. 2013; 8(9). doi: 10.1371/journal.pone.0075347 PMID: WOS:000324777300073.
22. Guerfal M, Ryckaert S, Jacobs PP, Ameloot P, Van Craenenbroeck K, Derycke R, et al. The HAC1 gene from *Pichia pastoris*: characterization and effect of its overexpression on the production of secreted, surface displayed and membrane proteins. Microb Cell Fact. 2010; 9. doi: 10.1186/1475-2859-9-49 PMID: WOS:000280174400001.
23. Xiong AS, Yao QH, Peng RH, Han PL, Cheng ZM, Li Y. High level expression of a recombinant acid phytase gene in *Pichia pastoris*. J Appl Microbiol. 2005; 98(2):418–28. doi: 10.1111/j.1365-2672.2004.02476.x PMID: WOS:000226398500018.
24. Yan JY, Zheng XL, Li SY. A novel and robust recombinant *Pichia pastoris* yeast whole cell biocatalyst with intracellular overexpression of a *Thermomyces lanuginosus* lipase: Preparation, characterization and application in biodiesel production. Bioresource Technol. 2014; 151:43–8. doi: 10.1016/j.biortech.2013.10.037 PMID: WOS:000330085800007.

25. Tashiro K, Nakamura T, Honjo T. The signal sequence trap method. Method Enzymol. 1999; 303:479–95. PMID: WOS:000081913000027.

26. Cereghino JL, Cregg JM. Heterologous protein expression in the methylotrophic yeast *Pichia pastoris*. Fems Microbiol Rev. 2000; 24(1):45–66. doi: 10.1111/j.1574-6976.2000.tb00532.x PMID: WOS:000084915900004.

27. Basco RD, Cueva R, Andaluz E, Larriba G. In vivo processing of the precursor of the major exoglucanase by KEX2 endoprotease in the *Saccharomyces cerevisiae* secretory pathway. Bba-Mol Cell Res. 1996; 1310(1):110–8. doi: 10.1016/0167-4889(95)00156-5 PMID: WOS:A1996TR16900017.

28. Schechter I, Berger A. On the size of the active site in proteases. I. papain (Reprinted fromBiochemical and Biophysical Research Communications, vol 27, pg 157, 1967). Biochem Bioph Res Co. 2012; 425(3):497–502. PMID: WOS:000308626200003.

29. Germain D, Thomas DY, Boileau G. Processing of Kex2 pro-region at two interchangeable cleavage sites. FEBS Lett. 1993; 323(1–2):129–31. PMID: 8495725.

30. Rholam M, Brakch N, Germain D, Thomas DY, Fahy C, Boussetta H, et al. Role of Amino-Acid-Sequences Flanking Dibasic Cleavage Sites in Precursor Proteolytic Processing—the Importance of the First Residue C-Terminal of the Cleavage Site. Eur J Biochem. 1995; 227(3):707–14. doi: 10.1111/j.1432-1033.1995.tb20192.x PMID: WOS:A1995QE85500014.

31. Edman P. Method for Determination of the Amino Acid Sequence in Peptides. Acta Chem Scand. 1950; 4(2):283–93. doi: 10.3891/acta.chem.scand.04-0283 PMID: WOS:A1950UW32800009.

32. Tschopp JF, Sverlow G, Kosson R, Craig W, Grinna L. High-Level Secretion of Glycosylated Invertase in the Methylotrophic Yeast, *Pichia-Pastoris*. Bio-Technol. 1987; 5(12):1305–8. doi: 10.1038/nbt1287-1305 PMID: WOS:A1987L112000020.

33. Ellis SB, Brust PF, Koutz PJ, Waters AF, Harpold MM, Gingeras TR. Isolation of alcohol oxidase and two other methanol regulatable genes from the yeast *Pichia pastoris*. Molecular and cellular biology. 1985; 5(5):1111–21. PMID: 3889590; PubMed Central PMCID: PMC366829.

34. Cregg JM, Madden KR, Barringer KJ, Thill GP, Stillman CA. Functional characterization of the two alcohol oxidase genes from the yeast *Pichia pastoris*. Molecular and cellular biology. 1989; 9(3):1316–23. PMID: 2657390; PubMed Central PMCID: PMC362724.

35. Cregg JM, Tschopp JF, Stillman C, Siegel R, Akong M, Craig WS, et al. High-Level Expression and Efficient Assembly of Hepatitis-B Surface-Antigen in the Methylotrophic Yeast, *Pichia-Pastoris*. Bio-Technol. 1987; 5(5):479–85. doi: 10.1038/nbt0587-479 PMID: WOS:A1987H071700018.

36. Chiruvolu V, Cregg JM, Meagher MM. Recombinant protein production in an alcohol oxidase-defective strain of *Pichia pastoris* in fedbatch fermentations. Enzyme Microb Tech. 1997; 21(4):277–83. doi: 10.1016/S0141-0229(97)00042-2 PMID: WOS:A1997XW35100007.

37. Brake AJ, Merryweather JP, Coit DG, Heberlein UA, Masiarz FR, Mullenbach GT, et al. Alpha-Factor-Directed Synthesis and Secretion of Mature Foreign Proteins in *Saccharomyces-Cerevisiae*. P Natl Acad Sci-Biol. 1984; 81(15):4642–6. doi: 10.1073/pnas.81.15.4642 PMID: WOS:A1984TF40900006.
